# A Concise Review on Current Trends in Imaging and Surgical Management of Hepatocellular Carcinoma

**DOI:** 10.7759/cureus.9191

**Published:** 2020-07-14

**Authors:** Joseph Asemota, Mohammed Saleh, Osato Igbinovia, Danny Burns

**Affiliations:** 1 Clinical Anatomy, St. George's University School of Medicine, True Blue, GRD; 2 Hematology/Oncology, Beth Israel Deaconess Medical Center, Boston, USA; 3 Internal Medicine, Howard University Hospital, Washington, USA; 4 Cancer Imaging, University of Hull, Hull, GBR; 5 Clinical Anatomy, St. George's University School of Medicine, St. George's, GRD

**Keywords:** imaging, liver transplant, hepatic resection, liver cirrhosis, management, hepatocellular carcinomas (hcc), gastroenterology, hepatology

## Abstract

Hepatocellular carcinoma (HCC) is a primary cancer of the liver whose incidence has seen an upsurge in the United States within the last 2 decades. Despite improvements in detection and management techniques, the prognosis for patients with HCC generally remains poor. There are multiple factors that have been implicated in the etiology of HCC with cirrhosis occurring as a common final pathway. This review presents a concise summary of current trends in imaging and surgical management of HCC. An internet-based (PubMed) search using the search terms “hepatocellular carcinoma” and “imaging” and "surgical management" was performed. Our search was limited to articles related to human studies published in English during the period of 07/01/2011 to 06/30/2016. A review of all relevant articles was conducted, and findings were summarized. Modern imaging modalities employed in the diagnosis of HCC include ultrasound scan (USS), computed tomography (CT), and magnetic resonance imaging (MRI) scan. The utility of diagnostic imaging is enhanced when interpreted in conjunction with appropriate laboratory tests such as alpha-fetoprotein.

The definitive treatment for HCC remains challenging; hepatic resection (HR) and liver transplantation (LT) are two approaches offering potentially curative options. For patients undergoing HR, important considerations include achieving maximum resection while maintaining optimal post-resection liver remnant volume (LRV) and functional capacity (FC), which can be assessed using 3-dimensional CT and indocyanine green clearance. Generally, an LRV of 40-50% is considered an acceptable lower limit for individuals with HCC compared to 20-30% among individuals with normal livers. With increasing knowledge of disease pathology, appropriate patient selection, coupled with advances in anesthesia and surgical technique, overall 5-year survival rates have significantly improved.

Challenges associated with LT on the other hand include donor-liver shortages with resultant long wait times and continued disease progression. The scarcity of cadaveric-donor livers has led to employing living-donor livers. Ethical considerations with respect to subjecting potentially healthy donors to undue morbidity and mortality risk however remain. Additional donor-shortage circumventing strategies include employing marginal, domino, and split-organ liver transplants. For patients awaiting transplant, employing bridging therapy such as radiofrequency ablation and transhepatic artery chemoembolization might occasionally help slow disease progression and maintain transplant eligibility. Appropriate patient selection achieved through the Milan and UCSF criteria designed to guide allotment of donor livers to patients with the best chances of survival could help improve outcomes and 5-year survival rates. The main radiological options for diagnosis include USS, CT, and MRI. HR and LT are two distinct surgical options, which in practice can be used to complement one another. Appropriate patient selection is necessary to achieve maximum benefits from HCC therapies.

## Introduction and background

Hepatocellular carcinoma (HCC) is a malignant disease arising from liver cells. It is the most common of the primary liver cancers and has a strong association with chronic exposure to aflatoxins and infection with tumorigenic pathogens, such as Hepatitis B virus and Hepatitis C virus [1]. HCC is one of the major causes of cancer morbidity and mortality worldwide. It is especially prevalent in developing countries where food contamination with aflatoxins and infections with Hepatitis B and C viruses are more common. However, its occurrence is beginning to increase in developed countries due to the high prevalence of diseases such as obesity and diabetes that can result in cirrhosis of the liver, one of the final common pathways in the pathogenesis of HCC [2]. 

Given the complexity in the pathogenesis and course of HCC, it is best managed by multidisciplinary teams comprising surgeons, diagnostic and interventional radiologists, oncologists, hepatologists, and pathologists. The HCC as well as its underlying causes will need to be treated or controlled. Multidisciplinary management helps in the individualization of treatment strategies, with the ultimate goal of optimizing patient outcomes. This concise review highlights the imaging and surgical treatment modalities available for HCC with the goal of aiding clinicians to make better informed individualized management decisions.

## Review

Diagnostic imaging

There are quite a number of diagnostic techniques that can be used in arriving at a diagnosis of HCC [3]. Upon suggestive clinical history and examination, the appropriate use of radiological imaging, laboratory tests, and pathological evidence from biopsies can aid in the accurate diagnosis of HCC [4].

The main radiological options include ultrasound scanning (USS), computed tomography (CT), and magnetic resonance imaging (MRI) scans with each modality having its specific advantages as well as limitations. 

Ultrasound Scan Imaging

**Figure 1:**
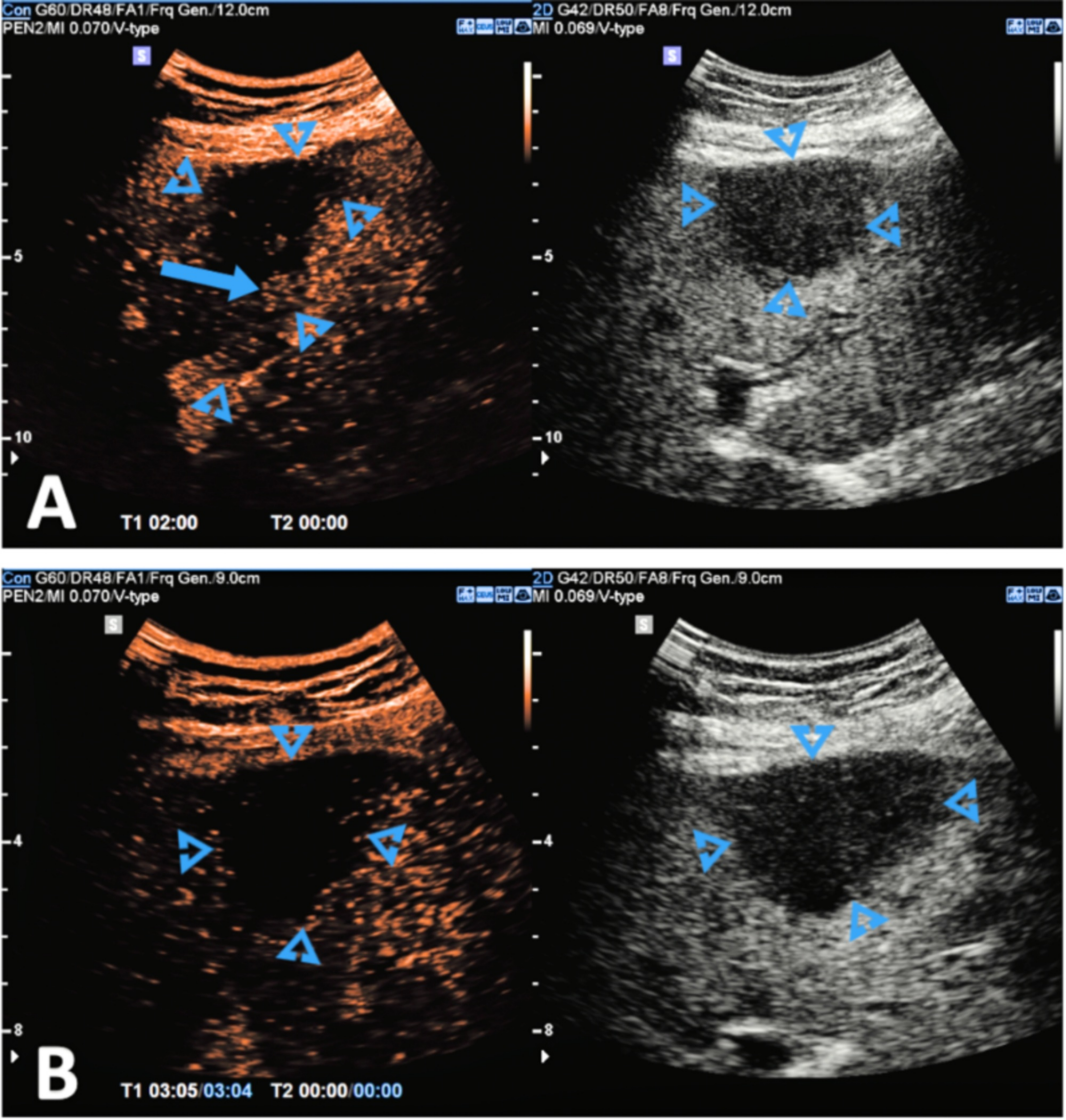
(A-B) After administration of IV An ultrasound contrast agent (SonoVue). The largest lesion is monitored throughout the arterial phase. The mass again shows internal vascularity with slightly delayed enhancement, followed by washout in the portal. Image Courtesy: Dr Balint Boltz, Radiopaedia.org, ID:70877; https://radiopaedia.org/cases/70877/studies/81075?lang=us IV, intravenous

**Figure 2:**
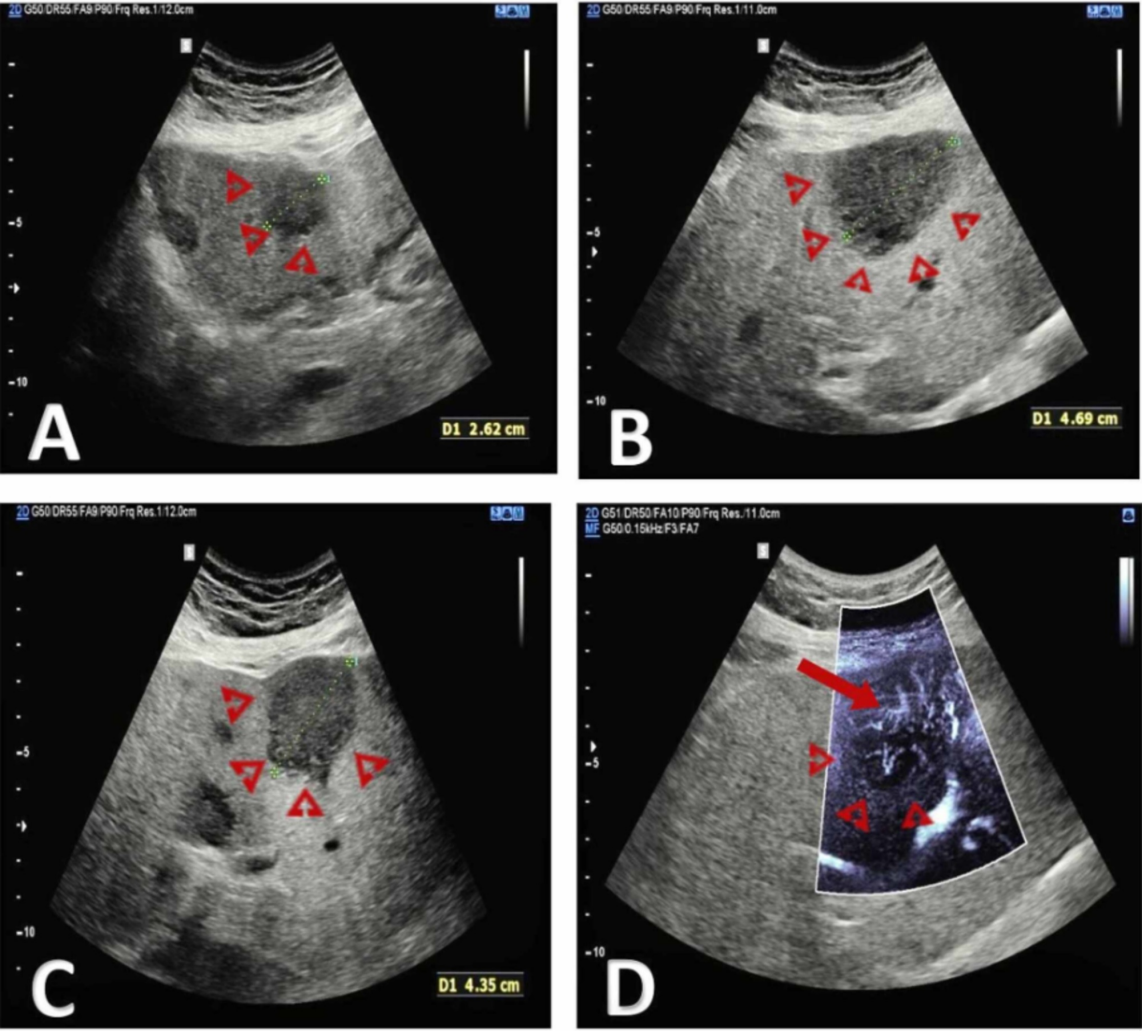
(A, B, C, and D) Multiple hypoechogenic solid masses are visible in the liver using B-mode ultrasound The lesions show increased internal vascularity when interrogated using superb microvascular imaging. Image courtesy: Dr Balint Botz, Radiopaedia.org, rID: 70877; https://radiopaedia.org/cases/recurrent-hepatocellular-carcinoma-ceus?lang=us

Ultrasound scanning is cost-effective and does not expose patients to ionizing radiation [5]. The American Association for the Study of Liver Disease (AASLD) recommends that high-risk patients undergo ultrasound screening every six months [6]. When a lesion of less than 1 cm is identified, it should be re-examined every three months. If the lesion increases in size, further evaluation with CT and/or MRI is indicated [7]. Major limitations to the use of ultrasound include its dependency on the operator’s skill, as well as its relatively low sensitivity and specificity with regard to the characterization of liver masses [8]. However, some studies have shown that the use of contrast-enhanced ultrasound studies can improve tumor characterization [9,10]. Additionally, contrast ultrasound guidance is particularly useful in improving the diagnostic accuracy of biopsy procedures [10]. In ultrasound contrast studies, HCC characteristically displays a vascular profile of enhancement during the arterial phase with washout during the venous phase [10] (Figures 1 and 2).

Computed Tomography Imaging

**Figure 3:**
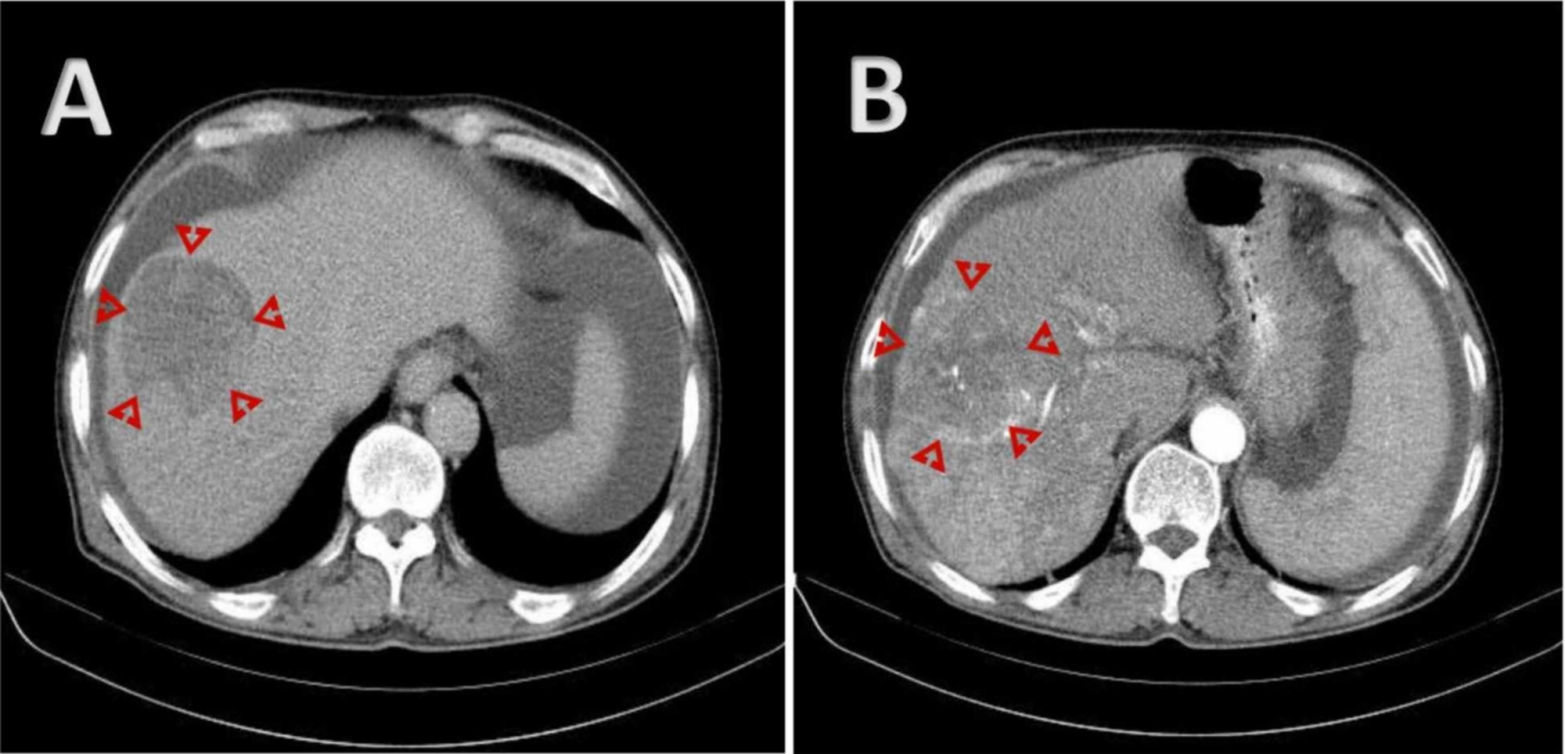
(A-B) CT imaging showing a mass in a cirrhotic liver with mosaic enhancement on early phases and rapid washout on delayed phase, compatible with hepatocellular carcinoma A: Axial C + arterial phase; B: Axial C + portal venous phase Image courtesy of Dr Mohammad Taghi Niknejad, Radiopaedia.org, rID: 20886; https://radiopaedia.org/cases/hepatocellular-carcinoma-with-portal-vein-tumour-thrombosis

Suspicious lesions identified in ultrasound are often further evaluated on CT. Commonly utilized CT scanning technologies include spiral CT and multi-detector CT, both of which enjoy significantly high specificity at about 93% [11-12]. The sensitivity of multi-detector CT is, however, higher at about 81% compared to about 68% for spiral CT [7,12]. The limitations of this imaging modality are patient exposure to ionizing radiation and low sensitivity (33.45%) for lesions less than 1 cm [8]. Nonetheless, its ability to allow for three-dimensional reconstruction images useful for operative planning - a capability not available with MRI despite its higher sensitivity and specificity - makes it very useful in the management of HCC patients [13]. Similar to its USS appearance, HCC characteristically demonstrates arterial phase enhancement and a venous phase washout with rim enhancement due to retention of contrast in its fibrous capsule [14] (Figure 3).

Magnetic Resonance Imaging

**Figure 4:**
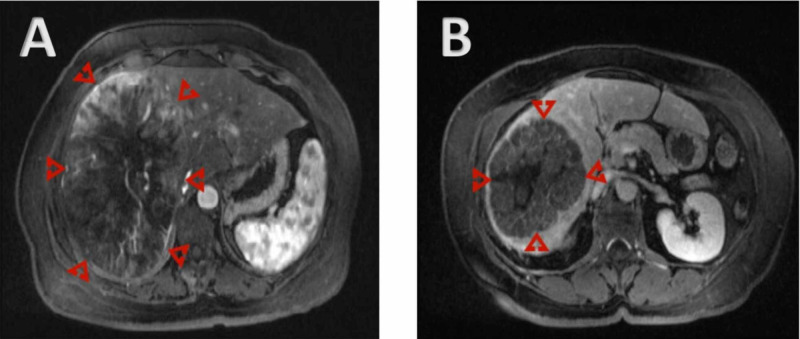
(A-B) MRI showing hypervascular liver mass with washout on delayed images A: Axial T1 FS C+ arterial phase; B: Axial T1 FS C+ delayed Case courtesy: Dr Ahmed Subaie, Radiopaedia.org, rID: 25953; https://radiopaedia.org/cases/hepatocellular-carcinoma-13?lang=us MRI, magnetic resonance imaging

Similar to the ultrasound and CT contrast studies, HCC demonstrates arterial phase enhancement and venous phase washout in MRI studies with contrast [15] (Fig 4). The commonly used gadolinium-based MRI contrast studies allow for the detection of lesions greater than 2 cm with a sensitivity of about 91% and specificity of about 95% [16]. Hepatocyte-specific contrast agents such as gadoxetate and dimeglumine are being developed which enhance the ability of MRI to detect HCC lesions that are less than 1 cm [15,17]. The non-usage of ionizing radiation, with its lesion detection capabilities, makes this imaging modality particularly attractive. The length of time required to complete the MRI studies may constitute a challenge for critically ill patients. 

Surgical treatment options

As of yet, there is no definitive curative treatment strategy for HCC although liver transplantation and hepatic resection, the two main surgical approaches, are potentially curative [18-19]. However, the risk of tumor recurrence still remains a major challenge [20].

Hepatic Resection

This procedure, which involves the surgical resection of cancer with appropriate tumor-free margins, requires an adequate functional reserve of the liver. Radical resection of hepatic parenchyma in patients with an inadequate functional reserve can result in post-operative liver failure [21]. 

The adequacy of the future remnant volume, its functional capacity, and surgical safety can be assessed using three-dimensional CT imaging and indocyanine green (ICG) clearance [22-23]. On CT imaging, the remnant liver volume can be estimated on slide sections and then integrated to give an approximate calculation of the future post-surgical remnant liver volume. A value of between 20% and 30% of the total liver volume is generally acceptable in patients with normal livers [22]. However, HCC is uncommon in patients with normal livers and is more often associated with a diseased liver. In this group, a post-surgical remnant liver volume of between 40% and 50% is considered safe [23].

The ICG clearance test gives an indication of liver function by calculating the fractional retention of ICG - a dye that is exclusively excreted in bile without any significant metabolism or enterohepatic circulation. In healthy individuals, the normal ICG retention fraction is about 10%. Values of less than 10% allow for resection of up to two-thirds of the total liver volume; patients with ICG values between 10% and 19% can potentially tolerate resections of about a third of their total liver volume; a value between 20% and 29% would allow for resection as much as only a sixth of total liver volume, while patients with values 30% and above might be able to safely tolerate only limited resection [8]. 

For patients determined to have low future remnant liver volume, preoperative portal vein embolization as described by Makuuchi *et al*. can be considered [23-24]. This procedure relies on the regenerative capacity of the liver. Occlusion of the portal vein supplying the region of the tumor will result in compensatory hypertrophy of the other regions and this can be as much as 40% [18]. The rate and ability to elicit a hypertrophic response to portal vein embolization gives a good indication of the future remnant liver function [25]. Thus, portal vein embolization can double as a dynamic stress test prior to hepatic resection as well as a method for increasing resectability. Even though there are some concerns about its stimulating growth of the existing tumor as part of compensatory hypertrophy, portal vein embolization with subsequent resection is currently the gold standard for cases of small hepatic remnant volume [26].

In addition to considerations of the future remnant liver volume, the intra-operative surgical approach has been shown in some studies to affect the cumulative survival rate with anatomical resection demonstrating significantly higher rates than non-anatomical resection [27]. The preferred anatomical approach is a segment-based resection that targets the removal of the tumor-affected liver with all intra-segmental portal vein branches as HCC may metastasize through the portal venous system [27]. However, the sub-segmental non-anatomical approach may be the only practical option when it is necessary to maintain an adequate future remnant liver volume. This more technically demanding approach may be optimized with the use of intra-operative ultrasound that will help in identifying possible missed tumors as well as improving the detection of vascular invasion.

Other current trends in hepatic resection include minimally invasive laparoscopic surgeries. Studies investigating laparoscopic hepatic resection and comparing them to open hepatic resections have found equivalent outcomes with the added benefit of decreased surgical stress, minimal water, and electrolyte disturbances, and less overall financial burden on the healthcare system [28-30]. However, the learning curve for younger surgeons to achieve proficiency with minimally invasive procedures suggest that surgeons with extensive experience using both open and laparoscopic approaches should be present at surgery [31-33].

To conclude, with current advances in anesthesiology, surgical techniques, and better knowledge of disease pathology, the perioperative morbidity and mortality risk associated with hepatic resection in HCC patients has been reduced significantly with five-year survival rates being as high as 50% [34-36]. 

Liver Transplantation

Transplantation is a surgical option for HCC patients who have tumors not amenable to hepatic resection [37]. It allows for wide tumor excision margins, removal of intrahepatic metastasis, management of underlying liver pathology, and is not limited by considerations of future liver remnant volume [7,18]. The major challenge associated with transplantation is a dearth of available donor livers to meet the high demand for transplantation [18-19]. The need to allot the available donor livers to patients with the best chances of survival has led to the development of several transplantation eligibility criteria algorithms. The two most commonly used are the Milan and UCSF criteria [3]. Under the Milan criteria, HCC patients with a solitary lesion of ≤5 cm in diameter or up to three lesions with each being ≤ 3cm in diameter are categorized as being eligible for transplantation [38]. Using these criteria for patient selection has significantly increased five-year survival to about 76%, comparable to results in patients undergoing liver transplant for indications other than HCC [38]. The UCSF criteria include patients with a single lesion of ≤6.5 cm or up to three lesions with each being ≤4.5 cm, with a cumulative diameter of ≤8cm. Studies have shown that there is no statistically significant difference in survival among those meeting the Milan criteria versus those exceeding the Milan criteria. In one study, patients meeting UCSF criteria but exceeding Milan criteria had a two-year survival of 86% (95% CI, 54% to 96%) [39]. These results suggest that the UCSF criteria may better predict acceptable post-transplant outcomes than the Milan criteria [39].

Even with the establishment of these criteria, most patients have to be on a waitlist for a considerable time period before getting a donor liver, during which time continued progression of the disease results in some patients becoming ineligible for transplantation. Accordingly, bridging treatment strategies including radiofrequency ablation, trans-hepatic artery chemo-embolization (TACE) and even hepatic resection may be necessary to avoid waitlist dropout [40]. 

In the United States, Model for End-Stage Liver Disease (MELD) exception points are used to reduce waitlist dropout by giving preference to Stage two HCC patients needing liver transplantation who meet the Milan Transplant Criteria [41].

Difficulties associated with the acquisition of sufficient cadaveric-donor livers and resulting long waitlist times have led certain centers, especially in Asia, to perform living-donor liver transplants. Meta-analysis studies comparing this procedure to the traditional cadaveric-donor liver procedure have shown similar overall survival rates [42]. However, this raises ethical issues about exposing living donors to such levels of morbidity/mortality risk without any direct benefits to their health. Other strategies also in use in Asia include the use of marginal livers, split-organ transplants, and domino livers, wherein the native explanted liver of a liver transplant recipient is transplanted into another patient [3,43].

## Conclusions

In conclusion, HCC is a complex disease with a clinical management strategy that involves consideration of multiplex factors in clinical decision-making. The surgical management strategies include hepatic resection and liver transplantation, which should not be considered using an either-or approach, but rather should be seen as complimentary tools along a spectrum. Just like most surgical procedures, appropriate patient selection remains a sine qua non for the demonstration of maximum benefit by these surgical strategies.
